# Cognitions about bodily purity attenuate stress perception

**DOI:** 10.1038/srep38829

**Published:** 2016-12-09

**Authors:** Kai Kaspar, Sarah Cames

**Affiliations:** 1 Department of Psychology, University of Cologne, Richard-Strauss-Str. 2, 50931, Cologne, Germany; 2 Faculty of Psychology and Neuroscience, Maastricht University, P.O. Box 616, 6200 MD Maastricht, Netherlands

## Abstract

Based on the assumption that physical purity is associated with a clean slate impression, we examined how cognitions about bodily cleanliness modulate stress perception. Participants visualized themselves in a clean or dirty state before reporting the frequency of stress-related situations experienced in the past. In Study 1 (*n* = 519) and Study 2 (*n* = 647) cleanliness versus dirtiness cognitions reliably reduced stress perception. Further results and a mediation analysis revealed that this novel effect was not simply driven by participants’ cognitive engagement in stress recall. Moreover, we found that participants’ temporal engagement in the recall of past stressful events negatively correlated with the amount of perceived stress, indicating an ease-of-retrieval phenomenon. However, a direct manipulation of the number of recalled stressful events in Study 3 (*n* = 792) showed the opposite effect: few versus many recalled events increased the perceived frequency of past stress-related situations. Overall, these novel results indicate an interesting avenue for future research on cognitively oriented stress reduction interventions, add to the literature on purity-related clean slate effects, and may help to better understand washing rituals in patients with obsessive-compulsive disorders.

The experience of stress is a key issue in health research. Stressful events can elicit manifold adaptive responses of the body that aim to maintain homeostasis^1^, but long-lasting stress exposition can have significantly negative effects on health^2^. Besides such a response-oriented perspective, cognitive approaches to stress highlight the critical role of the individual’s cognitive appraisal of potentially stressful events^3^. In fact, while the literature reveals a rather inconsistent pattern regarding the effects of stressful life events on health^4^, many researchers seem to agree that the subjective perception of the individual is the core determinant of stress experiences^5^,^6^,^7^. Correspondingly, Deckro *et al*.^8^ concluded that “many believe that at times stress is caused more by the way we think about a problem than by the problem itself” (p. 282). Thus, researchers and therapists are particularly interested in “the degree to which situations in one’s life are appraised as stressful” (ref. 5, p. 385). Not surprisingly, many intervention programs aim to increase one’s cognitive behavioral skills in order to foster an adequate cognitive coping with stressful situations^9^. We will outline in the next paragraphs why cognitions about one’s own bodily purity may be also a candidate to modulate stress perception in a positive manner.

In the last decade, psychological research suggested that the state of bodily purity is associated with a relief from mental burdens. In a first series of empirical studies, Zhong and Liljenquist^10^ found that the feeling of moral impurity, induced by recalling an unethical deed of the past, elicited a desire for body cleansing. In this sense, several hundred years ago, Shakespeare has already provided a beautiful description of this mechanism, as Lady Macbeth hoped that water could cleanse her of the murder of King Duncan. Accordingly, this effect has been called the “Macbeth effect” in the psychological literature. More recently, Gollwitzer and Melzer^11^ reported that inexperienced (versus frequent) players of games involving violence against humans felt higher moral distress. To cope with this uncomfortable state, they selected more hygiene products in a task on product preferences. Lee and Schwarz^12^ found that this desirability effect is coupled to the motor modality involved in a moral transgression: mouthwash was preferred after lying in a voice mail, whereas a hand sanitizer was preferred after lying in an email written by hand. Also, Denke *et al*.^13^ found that this desirability effect is accompanied by higher activity in a cortical network comprising sensorimotor areas. In line with these findings, it has also been shown that one’s willingness to voluntarily help another person was reduced after body cleansing^10^,^14^,^15^, indicating that bodily purity can remove moral stains and restore one’s moral self-image in a way that no further moral action appears to be necessary. Thus, body cleansing may be an effective strategy to cope with mental burdens.

According to Rozin *et al*.^16^, evolution adopted the mental circuitry initially developed for processing physical threats to deal with social threats. Similarly, Williams *et al*.^17^ argue that such effects can be explained when assuming that “early sensorimotor experiences serve as the foundation for the later development of more abstract concepts and goals” (p. 1257). In particular, we learn how to cleanse our body in early childhood and hence we build up a mental concept of bodily purity very early in life. Later in life, this knowledge may serve as a conceptual scaffold to understand the abstract concept of moral purity, resulting in an established conceptual link between body-related and abstract cognitions. As a consequence, “impressions about one’s own physical purity may affect conceptually related cognitions about morality” (ref. 18, p. 2). Importantly, activating this conceptual link does not require the bodily sensation of purity. Instead, it appears sufficient to cognitively activate the concept of bodily purity. This mechanism has been demonstrated by several researchers who found that the mere activation of cleanliness-related cognitions modulated judgments about morally relevant behavior^18^,^19^,^20^.

However, how could stress appraisal be linked to such effects related to the moral domain? Previous research also suggested that the effect of body cleansing is not limited to judgements in the moral domain. The Macbeth effect seems to be only a special form of a more general effect. Kaspar *et al*.^21^ showed that hand cleansing reduced the arousal of participants, as indicated by a decreasing pupil diameter. Moreover, Lee and Schwarz^22^ observed that hand cleansing reduced cognitive dissonance, while Florack *et al*.^23^ reported that hand washing reduced participants’ decision preferences biased by ownership. Similarly, Xu *et al*.^24^ found that the impression of good and bad luck can be metaphorically washed away as indicated by changes in one’s decision making strategy after hand cleansing. Finally, Kaspar^25^ found that hand washing increased participants’ optimism after they had experienced failure in a cognitive task. All these results indicate that cleansing of one’s own body does not only remove moral stains but that it helps to close a matter in a more general sense. Correspondingly, Lee and Schwarz^22^ labeled this phenomenon as the “clean slate effect”. It has been argued that purity-related cognitions can metaphorically remove traces of the past^24^, but an elaborated explanation of the effect is still missing. It might be that the clean slate effect is a signature of a learning process in which ritualized actions establish physical cleansing as a reset signal. Indeed, body cleansing is a widespread ritual often performed to finish a sequence of actions in everyday life (e.g., after working, after sport activities, or before we sleep). Thus, this daily routine is presumably associated with a strong clean slate impression, indicated by the reported attenuation of perceived mental burdens that derived from immoral behavior, failure, bad luck, or cognitive dissonance.

With respect to stress perception, we thus assumed that the impression of bodily purity can induce such a clean slate effect, reflected in a reduction of perceived stress. Of course, impressions of bodily purity do not remove external stressors in a literal sense, but they may be effective in modulating one’s cognitive appraisal of potentially stressful situations. Hence, purity-related cognitions may add a further cognitive strategy to the growing body of research suggesting “that cognitive appraisals are powerful tools that help shift negative stress states to more positive ones” (ref. 26, p. 51). Surprisingly, although research on stress appraisal and coping considers bodily responses to stressful events^27^,^28^,^29^, bodily purity, to our best knowledge, has not been related to stress perception so far. Thus, the present research question addresses on an almost blank page.

## Study 1

The aim of Study 1 was to demonstrate a potential clean slate effect in the context of stress perception. We expected that cleanliness cognitions, compared to dirtiness cognitions, would reduce perceived stress. Therefore, we compared two conditions in which participants visualized themselves in a clean or dirty state before they rated the amount of stress perceived within the last four weeks. We hypothesized:

**Hypothesis 1:** Cleanliness cognitions reduce the perceived stress compared to dirtiness cognitions.

### Method

*Participants.* The a priori estimation of the required sample size was based on a small effect (*d* = 0.30) found by Zhong *et al*. (ref. 20, Study 2), when applying a similar visualization task. Given a desired power of 0.80, a significance level of 0.05, and a two-tailed hypothesis testing, the required minimum sample size per condition was *n* = 176 (GPower software^30^). Overall, 519 participants (438 females) with a mean age of 26.76 years (*SD*_*age*_ = 7.86) had been randomly assigned to the cleanliness condition (*n* = 262, 220 females) or the dirtiness condition (*n* = 257, 218 females).

*Procedure.* We recruited the participants through databases of several German universities and through active promotion per emails and social networks. The participants were informed that the link will lead them to a research survey and that participation is voluntary. After clicking on the link to open the study, we informed the participants that their data will be processed anonymously and only for research purposes. Hence, written informed consent was not applicable to ensure participants’ anonymity. Instead, we informed the participants that they can stop the study whenever they want and that completion of the survey is considered to indicate informed consent. The studies and procedure were approved by the Research Ethics Board of Psychology & Neuroscience, Maastricht University. All procedures were carried out in accordance with the approved guidelines as well as the ethical guidelines of the German Psychological Society (DGPs).

At the beginning of the study, the participants provided their gender and age. Then, they performed a visualization task adapted from Zhong *et al*.^20^. This task required them to adopt a first-person perspective and to rate ten statements in which they visualized themselves in a clean or dirty bodily state. Participants rated each statement on a 7-point-scale ranging from 1 (*not at all*) to 7 (*very much*). In the cleanliness condition, participants were instructed to indicate how much they associate the following statements with physical cleanliness (English translations of German items): *My hair feels clean. My facial skin feels clean. My nose is clean. My breath is fresh. My clothes are freshly laundered. My hands are washed. My fingernails are freshly clipped. I smell of perfume/after shave. My feet are clean. I am freshly shaved*. Participants in the dirtiness condition were asked to rate how strong they associate the following statements with physical dirtiness: *My hair feels oily. My facial skin feels fatty. I need to clean and blow my nose. I have a bad breath. My clothes are dirty and sweaty. My hands feel sticky and dirty. My fingernails are too long. I am whiffy. My feet stink. I am unshaved*. After this visualization task, participants were asked to answer the German version of the Perceived Stress Questionnaire (PSQ^4^). This questionnaire is applicable for adults of any age, sex, occupation, and stage of life, and it consists of twenty statements describing unspecific stress-related situations (e.g., “*You have many worries*“, “*You feel mentally exhausted*“, “*You feel under pressure from deadlines*”, and “*You are full of energy*”). The participants rated on a 4-point scale, ranging from 1 (*almost never*) to 4 (*mostly*), how frequently they experienced these stress-related situations within the last four weeks. The mean score across all items was used as a valid indicator of the overall stress perception^4^, characterized by a high coefficient alpha reliability of 0.91.

### Results and discussion

To test the effect of the visualization condition on perceived stress, we calculated a *t*-test for independent samples. We found lower stress values in the cleanliness condition (*M* = 2.212, *SD* = 0.523) compared to the dirtiness condition (*M* = 2.301, *SD* = 0.483), *t*(517) = −2.012, *p* = 0.044, *d* = 0.177, 95% *CI*_*d*_ = 0.005 to 0.350. Hence, the cleanliness visualization reduced the perceived stress in comparison to the dirtiness visualization, supporting Hypothesis 1. Thereby, the extent to which participants agreed to the statements of the visualization task was not correlated with perceived stress in both the cleanliness condition, *r* = −0.054, *p* = 0.385, and the dirtiness condition, *r* = 0.046, *p* = 0.466.

Further, we examined the processing duration of the PSQ indicating the time participants required to assess the frequency with which they have experienced stress-related situations within the past four weeks. However, the data required some pre-processing due to few extreme outliers that would otherwise significantly bias the results. First, in order not to bias the mean across all participants, we provisionally excluded participants who showed a total processing duration of more than 300 seconds (7 of 519 participants). We then calculated the mean processing duration (*M* = 91.21) and the corresponding standard deviation (*SD* = 41.32). Next, 2.7% of all participants were identified as outliers as their processing duration was more than three standard deviations above the mean. However, following Custers and Aarts^31^, these participants were not removed but their processing durations were truncated to the mean value plus three standard deviations. This procedure preserved the maximum power for statistical testing. We found a mean processing duration of *M* = 87.94 seconds (*SD* = 38.47) after the cleanliness visualization and of *M* = 96.99 seconds (*SD* = 44.79) after the dirtiness visualization, *t*(502.477) = −2.467, *p* = 0.014, *d* = 0.217, 95% *CI*_*d*_ = 0.044 to 0.389. Thus, cleanliness cognitions reduced the time participants thought about stress-related situations that they had experienced within the past four weeks. Apparently, cleanliness cognitions, compared to dirtiness cognitions, did not only reduce the perceived stress but they also reduced participants’ temporal engagement in the assessment of past stress-related situations. This result pattern supports the notion of a clean slate effect according to which cleanliness can alleviate or even remove memory traces of past events^24^.

As an important side note, participants’ temporal engagement in processing the PSQ did not influence the internal reliability of the stress scale. We divided the sample into two subsamples by using the median completion time of the PSQ (80 seconds). Participants who spent less time in filling out the stress scale showed an even slightly higher coefficient alpha reliability (α = 0.92) compared to participants who spent more time (α = 0.90).

We also investigated the visualization task in more detail. We applied the truncation procedure described above to the visualization duration: we provisionally excluded nine participants who showed a visualization duration of more than 300 seconds; we then calculated the mean visualization duration (*M* = 61.23) and standard deviation (*SD* = 36.31); finally, 3.9% of all participants were identified as outliers and their visualization durations were truncated to the mean value plus three standard deviations. We observed a significant group difference regarding the visualization duration, with a mean duration of *M* = 58.41 seconds (*SD* = 31.52) for the cleanliness condition and of *M* = 65.85 seconds (*SD* = 37.54) for the dirtiness condition, *t*(498.644) = −2.443, *p* = 0.015, *d* = 0.215, 95% *CI*_*d*_ = 0.042 to 0.387. Hence, the participants were longer involved in the visualization task when they visualized themselves in a dirty bodily state. We speculate that while the cleanliness statements may have exclusively triggered cognitions related to cleanliness, the dirtiness statements may have triggered dirtiness and cleanliness cognitions, because when judging the dirtiness of clothes or hands (*My clothes are dirty and sweaty, My hands feel sticky and dirty*), one may additionally need to visualize a “clean reference level”. This process may explain why participants spent more time when they visualized themselves to be dirty versus clean. Moreover, the data showed that having a clean versus fatty facial skin, for example, was not perceived as similarly significant for one’s bodily cleanliness versus dirtiness: the participants agreed more with the cleanliness statements (coefficient alpha reliability: α = 0.84; *M* = 5.31, *SD* = 0.90) compared to the dirtiness statements (α = 0.91; *M* = 4.14, *SD* = 1.44), *t*(429.158) = 11.140, *p* < 0.001, *d* = 0.978, 95% *CI*_*d*_ = 0.796 to 1.160. Hence, we may assume that the present cleanliness and dirtiness statements did not describe similarly pronounced ends of a bipolar construct. Instead, gradations of cleanliness might map onto a unipolar dimension, whereby cleanliness serves as a rather unambiguous reference level. In fact, the smaller standard deviation observed for the cleanliness versus dirtiness statements (Levene’s test: *F* = 61.425, *p* < 0.001) indicates that the cleanliness statements left less room for inter-individual differences in interpretation.

Additionally, asking about bodily cleanliness/dirtiness could have different effects depending on a person’s current bodily cleanliness/dirtiness. This might actually produce a contrast effect where dirty people are even more engaged in dirtiness cognitions after answering cleanliness statements. Such contrast effects may increase the noise in the data and reduce the power of the manipulation. Nonetheless, this treatment was effective in the present Study 1 as well as in a previous one^18^.

All in all, Study 1 made a first attempt to examine the impact of cleanliness and dirtiness cognitions on stress perception. In accordance with Hypothesis 1 and the notion of a clean slate effect associated with bodily purity, we found that cleanliness cognitions reduced the perceived frequency of stress-related situations experienced in the recent past. Also, the cleanliness group took less time to assess the frequency of past stress-related situations. We may speculate that the effect of cleanliness/dirtiness cognitions on stress perception was (at least partially) mediated by one’s willingness to engage in an elaborate recall of past stressful events. We hence pursued this potential mediation pathway in Study 2. However, the visualization task in Study 1 might have elicited a contrast effect where dirty people were even more engaged in dirtiness cognitions after answering cleanliness statements. Thus, we slightly changed the visualization task in Study 2 to examine the robustness of its effect on stress perception.

## Study 2

Study 2 aimed to replicate the cleanliness/dirtiness effect found in Study 1 and to shed first light on the mechanism behind it. This appears an important step as several previous studies in this field revealed mixed results. On the one hand, several studies failed to replicate the classic Macbeth effect in US samples^32^, in Spanish samples^33^, and a sample from the UK^34^. Moreover, Kaspar and Teschlade^18^ found different effects of a cleanliness versus dirtiness visualization task on morality ratings compared to Zhong *et al*.^20^. Further, Johnson *et al*.^35^ could not replicate a previously reported effect of cleanliness cognitions on moral judgments^19^, while differences in the subject populations has mentioned as one potential reason^36^. Such failures of replication challenge the general validity of reported effects. However, as pointed out recently^37^, “failed replications in this field might not always be a signature of biased results in original studies” but instead they might reflect “hitherto undiscovered moderation and mediation processes” (p. 8). Indeed, a recent study^18^ revealed an indirect effect of a cleanliness manipulation on one’s self-ranked moral character in the absence of any direct or total effect. Therefore, we decided to further examine participants’ engagement in the retrieval of past stressful events from memory (stress recall duration) that might have mediated the effect of cleanliness/dirtiness cognitions on perceived stress.

In general, previous studies showed that longer response times represent the processing of more context-relevant information stored in memory^38^,^39^ and that participants think longer about an issue to give a more complete response^40^. Moreover, it has been claimed that the impression of bodily purity systematically biases information processing^21^; that is, the clean slate effect associated with physical purity may represent a general attenuation of the perceived salience of past events, as indicated by several studies^22^,^23^,^24^,^25^. Accordingly, we hypothesized that cleanliness (versus dirtiness) cognitions would lead to a shorter stress recall duration, indicating a reduced cognitive elaboration of past stressful events, which, in turn, should reduce the sense that one has experienced stress frequently in the past. We thus formulated a corresponding mediation hypothesis:

**Hypothesis 2:** The temporal engagement in the retrieval of past stressful events from memory (stress recall duration) mediates the effect of a cleanliness/dirtiness visualization task on perceived stress.

### Method

*Participants.* In Study 2, 647 participants with a mean age of 26.72 years (*SD*_*age*_ = 7.32) had been randomly assigned to the cleanliness condition (*n* = 326, 249 females) or to the dirtiness condition (*n* = 321, 256 females). The recruiting strategy was the same as in Study 1.

*Procedure.* The procedure and materials were the same as in Study 1 with exceptions noted. After the participants had provided their age and gender, they were randomly assigned to one of the two visualization conditions. The participants were again presented the identical visualization items used in Study 1. However, this time their task was to carefully read each statement and to imagine that the bodily state described currently applies to them and how it feels like. This change in the manipulation was intended to avoid the potential contrast effect discussed in Study 1. After this visualization task, the participants had to indicate on a 7-point scale (1 = *not at all*; 7 = *very*) to which extent they agree with the following statement: “*I feel bodily clean/dirty in the imagined state*”. This item served as a manipulation check. On the next page of the online study, we asked the participants to carefully recall all stressful events they had experienced within the past four weeks. The participants had to click on a control checkbox before they could continue with the next page presenting the perceived stress questionnaire (PSQ). Thus, the participants had been required to recall *concrete* stressful events before they assessed the frequency of different but more unspecific stress-related situations experienced in the past, such as time pressure, trouble in relaxing, and mental exhaustion. This second change in the procedure allowed us to test the new mediation hypothesis (Hypothesis 2).

### Results and discussion

First of all, we checked whether the cleanliness/dirtiness visualization task led to a different evaluation of participants’ imagined bodily state. As intended, the participants felt significantly cleaner in the cleanliness condition (*M* = 6.10, *SD* = 1.38) compared to the dirtiness condition (*M* = 1.47, *SD* = 1.08), *t*(612.325) = 47.609, *p* < 0.001, *d* = 3.736, 95 % *CI*_*d*_ = 3.481 to 3.992.

With respect to Hypothesis 1, we found higher stress values in the dirtiness condition (*M* = 2.51, *SD* = 0.56) compared to the cleanliness condition (*M* = 2.41, *SD* = 0.57), *t*(645) = 2.132, *p* = 0.033, *d* = 0.168, 95% *CI*_*d*_ = 0.013 to 0.322. Thus, we replicated the main effect found in Study 1 with a slightly different visualization task, indicating some robustness of the effect.

Moreover, we observed an increase of the stress scores in absolute terms. To test this statistically, we calculated a 2 (study: Study 1 versus Study 2) × 2 (visualization condition: cleanliness versus dirtiness) ANOVA with perceived stress as dependent variable. Indeed, the stress scores were significantly higher in Study 2, *F*(1, 1162) = 41.889, *p* < 0.001, *η*_*p*_^*2*^ = 0.035. We also found the effect of the visualization condition, *F*(1, 1162) = 8.416, *p* = 0.004, *η*_*p*_^*2*^ = 0.007, but the effect size should be interpreted with caution here as the treatment was different in the two studies. No interaction existed, *F*(1, 1162) = 0.006, *p* = 0.937, *η*_*p*_^*2*^ < 0.001. Hence, the difference between Study 1 and 2 in perceived stress was similarly pronounced in both visualization conditions. The higher stress scores in Study 2 suggest that explicitly recalling stressful events of the past before rating the items of the PSQ led to an increased stress perception in general.

In the next step, we tested the mediation hypothesis (Hypothesis 2). We assumed that the stress recall duration mediated the effect of the visualization condition on perceived stress. We applied an analysis of indirect effects according to Preacher and Hayes^41^, providing robust estimates for the indirect effect of the predictor variable on the outcome variable through mediators by means of a bootstrapping procedure (20,000 bootstrap samples, bias-corrected). The visualization condition served as the predictor variable (dummy-coded: 0 = clean, 1 = dirty), the stress recall duration as the mediator variable, and the perceived stress served as the outcome variable. As in Study 1, in order not to bias the results of the mediation analysis, we initially truncated the values of few outliers who showed extreme stress recall durations: we provisionally excluded seven participants (1%) who showed a stress recall duration of more than 100 seconds; we then calculated the mean duration (*M* = 23.61) and standard deviation (*SD* = 14.41); finally, 3.1% of all participants were identified as outliers and their stress recall durations were truncated to the mean value plus three standard deviations. As shown in Table 1, the effect of the visualization condition on perceived stress was a direct one. The dirtiness visualization led to higher stress scores while this effect was not mediated by the time participants were engaged in recalling concrete stressful events of the past. Interestingly, a short (versus long) stress recall duration increased the estimated frequency of stress-related situations experienced in the past. This negative correlation may represent another example of the well-known ease-of-retrieval effect^42^,^43^,^44^,^45^. As suggested by Tversky and Kahneman’s^46^ availability heuristic, people tend to estimate the frequency of an event as a function of the ease with which it comes to mind. That is, the briefer one thinks about an issue at the expense of an exhaustive recall, the easier becomes the recall process and the higher is the assessed number of events that could be recalled. With respect to the present study, a temporally short (but relatively easy) recall of past stressful events, compared to a more exhaustive recall of stressful events, increased the subsequently estimated frequency of stress-related situations experienced in the past four weeks.

Finally, we examined the processing duration of the PSQ. We applied the same truncation procedure as applied in Study 1 to 2.9% of participants. Surprisingly, we found a mean processing duration of *M* = 96.12 seconds (*SD* = 37.93) in the cleanliness group and of *M* = 90.26 seconds (*SD* = 33.19) in the dirtiness group, *t*(636.228) = 2.091, *p* = 0.037, *d* = 0.164, 95% *CI*_*d*_ = 0.010 to 0.319. This time, cleanliness cognitions prolonged the processing time of the PSQ, whereas the reverse effect was found in Study 1. Note that this original effect was the starting point of our mediation hypothesis not supported by the data of Study 2. Apparently, the reverse effect observed in Study 2 provides further counterevidence against the notion that the effect of cleanliness cognitions on stress perception is driven by a reduced willingness to think about past stressful experiences.

To conclude, we replicated the main effect of cleanliness/dirtiness cognitions on perceived stress. The mediation analysis showed that this effect was direct and not driven by participants’ temporal engagement in the retrieval of stressful events from memory. This result was further supported by a longer processing duration of the PSQ in the cleanliness group. Further, the data suggested an ease-of-retrieval effect as a short (versus long) stress recall duration increased the perceived frequency of past stress-related situations. However, some authors suggest a double randomization to investigate a mediation hypothesis^47^,^48^. In such an experimental design, participants are not only randomly assigned to the levels of the independent variable to examine its impact on the observed mediator. Rather, participants are also randomly assigned to different levels of the mediator to examine its effect on the observed dependent variable. Although we did not find an effect of the independent variable (visualization condition) on the mediator (stress recall duration), an experimental manipulation of the mediator was nonetheless recommended to further investigate the relationship between participants’ engagement in stress recall and perceived stress (dependent variable). In the final Study 3, we aimed to clarify whether a longer temporal engagement in the retrieval of past stressful events from memory actually reflected the recall of more stressful events, or whether it rather reflected prolonged rumination about individual events. We therefore asked participants to explicitly write down stressful events they recall.

## Study 3

Given the negative correlation between stress recall duration and perceived stress found in Study 2, and in accordance with evidence for ease-of-retrieval effects in different domains^42^,^43^,^44^,^45^, we examined the following hypothesis in Study 3:

**Hypothesis 3:** The explicit recall of few (easy recall) versus many (difficult recall) stressful events of the past increases the perceived frequency of experienced stress-related situations.

### Method

*Participants.* In Study 3, the data of 792 participants with a mean age of 26.02 years (*SD*_*age*_ = 6.78) were analyzed. 441 participants (338 females) were assigned to the easy recall condition and 351 participants (288 females) were assigned to the difficult recall condition.

*Procedure.* The participants initially provided their age and gender. Afterwards, they were randomly assigned to one of two recall conditions. Following previous studies^42^,^49^,^50^, the participants were asked to recall and write down three (*easy recall*) or seven (*difficult recall*) different events in which they had experienced stress within the past four weeks. Participants were only considered in the data analysis when they provided the required number of different events. Finally, they filled out the PSQ.

### Results and discussion

We first examined the stress recall task that preceded the PSQ. For this purpose, we excluded 15 participants who invested more than 300 seconds into the easy or difficult recall task as they were considered as extreme outliers, undermining the manipulation. Importantly, the average time participants took to recall and write down three stressful situation (*M* = 94.77, *SD* = 38.21) or seven stressful situation (*M* = 93.50, *SD* = 39.64) did not differ, *t*(775) = 0.452, *p* = 0.651, *d* = 0.033, 95% *CI*_*d*_ = −0.109 to 0.174. Consequently, the manipulation did not affect participants’ temporal engagement in the retrieval of stressful events from memory but only the number of recalled stressful events.

We then examined Hypothesis 3 according to which we expected more perceived stress after recalling few versus many stressful events of the past. However, we found higher stress values in the difficult recall condition (*M* = 2.60, *SD* = 0.55) compared to the easy recall condition (*M* = 2.50, *SD* = 0.55), *t*(790) = 2.415, *p* = 0.016, *d* = 0.173, 95% *CI*_*d*_ = 0.032 to 0.313. This effect did not change when excluding the above-mentioned 15 outliers (*p* = 0.017).

Consequently, the present results suggest that the negative correlation between stress recall duration and perceived stress found in Study 2 was a temporal phenomenon. A longer stress recall duration apparently reflected prolonged rumination about individual events but did not reflect more events retrieved from memory. In contrast, when directly manipulating the number of recalled stressful events (while not changing the total temporal engagement), few versus many recalled events increased the perceived frequency of past stress-related situations.

## General Discussion

The present studies aimed to answer whether cognitions about one’s own cleanliness affect the amount of perceived stress. In Study 1 we found that cleanliness cognitions, compared to dirtiness cognitions, attenuated stress perception. With a slightly different treatment, we replicated this novel effect in Study 2. Hence, the results indicate some robustness of the effect. Study 1 also showed that the cleanliness group took less time to assess the frequency of past stress-related situations, whereas in Study 2 the effect was reversed. Moreover, participants’ temporal engagement in the retrieval of past stressful events from memory did not mediate the effect of cleanliness/dirtiness cognitions on perceived stress, as revealed by an analysis of indirect effects conducted in Study 2. Consequently, this novel effect seems to be not only a signature of one’s cognitive engagement in a stress recall task.

However, we found that participants’ temporal engagement in the retrieval of past stressful events from memory negatively correlated with the amount of perceived stress. Study 3 showed that this correlation was a temporal phenomenon, whereby a longer stress recall duration apparently reflected prolonged rumination about individual events but did not reflect more events retrieved from memory. A direct manipulation of the number of recalled stressful events showed the opposite effect: few versus many recalled events increased the perceived frequency of past stress-related situations.

Although the cleanliness/dirtiness visualization task slightly differed between Studies 1 and 2, the effect sizes observed were equally small. One might argue that the effect is too small to be of practical significance. However, we suggest to consider the present effects as the lower bound of a scale as far as no contrary evidence is available. Indeed, the present online studies realized a very short treatment and due to the unstandardized setting across participants, we were not able to control for participants’ actual engagement in the study or environmental distractions during responding, a common limitation of online research (cf.^51^). These aspects may have reduced the effectiveness of the treatment. It is conceivable that standardized conditions and a more intensive cleanliness/dirtiness treatment (or repeated treatments) can have more pronounced effects on stress perception, making the present account an interesting venue for future research on cognitively oriented stress reduction interventions. However, reducing the perceived salience of stressful events by using a body-related cleanliness treatment might also lead to an underestimation of what is actually harmful in the long run, such as continuous time pressure due to unrealistic deadlines. Consequently, potential negative effects should be beared in mind.

Moreover, it may be that real body cleansing, compared to the visualization task applied here, also leads to more pronounced effects because real cleansing is presumably more intensive and may elicit a stronger activation of cleanliness cognitions. At least with respect to cleanliness effects on moral judgments, real hand cleansing showed stronger effects^18^,^19^,^20^. In this context, it is noteworthy that Barsalou^52^ and Hesslow^53^ argue that the cognitive simulation of physical actions and states (such as body cleansing) can have the same consequences for brain responses as normally elicited by the corresponding overt behavior. According to Barsalou^52^, such cognitive “simulation is the reenactment of perceptual, motor, and introspective states acquired during experience with the world, body, and mind” (p. 618). Consequently, we may speculate that a corresponding visualization task can be similarly effective as real body cleansing. However, it has to be noted that the core mechanisms of observable phenomena in this field may be very different even in the case of comparable results at the phenomenological level. While a visualization task may trigger cleanliness cognitions that, in turn, prime conceptually related cognitions of a clean slate, the same effect produced by real body cleansing may be a signature of embodied cognition influencing stress perception in a direct way without the need of a “translation” into abstract cognitions about bodily purity (cf.^54^).

In each case, the present results are also of interest from a clinical perspective as they may add further information about potential causes of obsessive-compulsive disorders (OCD) with hand washing as primary symptom. As indicated by subjective reports, OCD patients perceive a stronger release from mental burdens after body cleansing^55^. They also showed a stronger decrease in the willingness to voluntarily help another person when they had cleansed their hands (versus no cleansing) after recalling an unethical deed of the past^56^. Hence, in the light of the present results, we may assume that OCD patients tend to perform body cleansing to produce a clean slate effect and hence to alleviate stressful traces of the past. Of course, this line of arguments has to be validated by much more research, but the present results add to the young and ongoing debate about the generalizability of purity-related clean slate effects and their boundary conditions. In a nutshell, cleanliness versus dirtiness cognitions reliably reduced perceived stress, indicating that the clean slate effect also generalizes to the domain of stress perception.

## Additional Information

**How to cite this article:** Kaspar, K. and Cames, S. Cognitions about bodily purity attenuate stress perception. *Sci. Rep.* **6**, 38829; doi: 10.1038/srep38829 (2016).

**Publisher's note:** Springer Nature remains neutral with regard to jurisdictional claims in published maps and institutional affiliations.

## Figures and Tables

**Table 1:** Results of the mediation analysis.

Model	Coefficient	SE	t	p
Model summary	R^2^ = 0.019, F(2, 644) = 6.233, p = 0.002**			
Effect of visualization condition (VC) on mediator	0.259	1.327	0.195	0.846
Direct effect of mediator on stress perception	−0.004	0.001	−2.806	0.005**
Total effect of VC on stress perception	0.094	0.044	2.132	0.033*
Direct effect of VC on stress perception	0.095	0.044	2.165	0.031*
Indirect effect of VC on stress perception through mediator	effect = −0.001 (SE = 0.005), 95%CI: −0.011 to 0.010			

*p < 0.05, **p < 0.01.
